# Optogenetic silencing of locus coeruleus activity in mice impairs cognitive flexibility in an attentional set-shifting task

**DOI:** 10.3389/fnbeh.2015.00286

**Published:** 2015-11-04

**Authors:** Kathrin Janitzky, Michael T. Lippert, Achim Engelhorn, Jennifer Tegtmeier, Jürgen Goldschmidt, Hans-Jochen Heinze, Frank W. Ohl

**Affiliations:** ^1^Department of Neurology, University of MagdeburgMagdeburg, Germany; ^2^Systems Physiology of Learning, Leibniz Institute of NeurobiologyMagdeburg, Germany; ^3^Center for Behavioral Brain SciencesMagdeburg, Germany; ^4^Systems Physiology, Institute of Biology, University of MagdeburgMagdeburg, Germany

**Keywords:** optogenetics, halorhodopsin, B6.Cg-Tg(Th-cre)1Tmd/J hemizygous mice, locus coeruleus, attentional set-shifting task, cognitive flexibility, extra-dimensional set-shifting, prefrontal cortex

## Abstract

The locus coeruleus (LC) is the sole source of noradrenergic projections to the cortex and essential for attention-dependent cognitive processes. In this study we used unilateral optogenetic silencing of the LC in an attentional set-shifting task (ASST) to evaluate the influence of the LC on prefrontal cortex-dependent functions in mice. We expressed the halorhodopsin eNpHR 3.0 to reversibly silence LC activity during task performance, and found that silencing selectively impaired learning of those parts of the ASST that most strongly rely on cognitive flexibility. In particular, extra-dimensional set-shifting (EDS) and reversal learning was impaired, suggesting an involvement of the medial prefrontal cortex (mPFC) and the orbitofrontal cortex. In contrast, those parts of the task that are less dependent on cognitive flexibility, i.e., compound discrimination (CD) and the intra-dimensional shifts (IDS) were not affected. Furthermore, attentional set formation was unaffected by LC silencing. Our results therefore suggest a modulatory influence of the LC on cognitive flexibility, mediated by different frontal networks.

## Introduction

The Attentional Set Shifting Task (ASST) as an animal analog of the ID/ED task was designed to dissociate between two categories of frontocortical based kinds of behavioral flexibility in rodents: reversal learning and set shifting (Birrell and Brown, 2000). The ASST requires animals to initially learn a rule and form an “attentional set” within the same stimulus dimension before an extra-dimensional shift (EDS) is performed. During the EDS mice have to switch their attention to a previously irrelevant dimension (Kos et al., 2011). Here we used a modified version of the ASST (Bissonette et al., 2008) for reliable set shifting in mice. Since attentional set shifting depends on successful prior formation of an attentional set, the task was designed with internal construct validation (Young et al., 2010) by recording the performance as a ratio of EDS and last IDS trials. If this ratio exceeds one, sufficient set formation can be assumed (Garner et al., 2006; Bissonette et al., 2008).

Regulation of attention and behavioral flexibility are important functions attributed to the networks of the prefrontal cortex (PFC). There is evidence for a distribution of different cognitive functions measured by the ASST within specific regions of the PFC. In rodents for example, reversal learning, when reinforcement contingencies are altered within a single stimulus domain, recruits and engages the orbitofrontal cortex (OFC), whereas attentional set-shifting, in which attention is reallocated to a previously irrelevant perceptual dimension, depends on the medial prefrontal cortex (mPFC; Hamilton and Brigman, 2015).

Prefrontal networks are strongly modulated by catecholamines. In particular there is a dense noradrenergic innervation of the PFC by the locus coeruleus (LC), a small nucleus in the brainstem that is the sole source of norepinephrine (NE) in the cortex (Aston-Jones and Cohen, 2005). NE in the PFC seems to be required for cognitive flexibility (Milstein et al., 2007; McGaughy et al., 2008; Chamberlain and Robbins, 2013). In situations that warrant to disengage attention from a previously relevant dimension that lost its relevance, the PFC is required for rapid and successful adaptation of behavior in the changing environment (Lapiz and Morilak, 2006; Tait et al., 2007).

Lesion studies have shown that noradrenergic deafferentiation of the mPFC caused selective impairment of EDS performance (McGaughy et al., 2008), and therefore support a special role for cortical NA in cognitive flexibility, when the animal shifts from attending from one perceptual dimension to another (Lapiz and Morilak, 2006; Lapiz et al., 2007; Tait et al., 2007; Desteno and Schmauss, 2008; McGaughy et al., 2008). Since those prior studies are based on irreversible lesion techniques or long lasting pharmacological manipulations they were unable to differentiate between effects on the acquisition phase, memory consolidation phase or even long term plastic changes. Considering that small changes of catecholaminergic activity in the PFC profoundly affect cognitive functions, the current study applied optogenetic LC silencing during brief periods of task performance in Th::Cre-mice to investigate the effects on learning new strategies independent from memory consolidation. Optogenetic silencing can be restricted to the acquisition phase alone and does not interfere with the functions of the LC during the remaining time. Since the use of TH-Cre mice allows to target TH-positive neurons of the LC in a highly specific manner and since microbial halorhodopsins enable the inhibition of those neurons on a short time scale with excellent reversibility, optogenetic methods are well suited to study the role of the LC on complex behavior (Carter et al., 2010; de Lecea et al., 2012). In summary, we used short-term optogenetic LC silencing and studied the effects on cognitive flexibility in the attentional set-shifting task.

## Materials and methods

### Animals

For the experiments we used naive male B6.Cg-Tg(Th-cre)1Tmd/J hemizygous mice purchased from Jackson Laboratories, *n* = 14, aged 10 weeks at the date of surgery. These mice express Cre-recombinase under the control of the tyrosine hydroxylase (TH) promoter and therefore in the noradrenergic neurons of the LC. All procedures were committed by the European Council guideline 86/609/EEC and approved by local authorities.

### Surgery and optogenetic transduction

Virus injection and fiber implantation were performed as described previously (Carter et al., 2010). Briefly, mice were anesthetized, and one hole was drilled above the LC (AP 5.5 mm, ML −0.85 mm). A pulled glass pipette was lowered to the depth of 3.7 mm (relative to Bregma) and 500 nl virus solution (AAV2-Ef1a-DIO-eNpHR 3.0-EYFP, generously provided by Karl Deisseroth through the UNC vector core, 1.5E12 particles/ml) were injected into the tissue at a rate of 50 nl/min. The virus was allowed to diffuse into the tissue for an additional 10 min after the end of injection, before the pipette was removed. Immediately afterwards, an optic fiber (220 μm, 0.37 NA, Doric Lenses) was implanted 400 μm above the center of the injection. We transduced the LC unilaterally to avoid overly strong effects of bilateral silencing on general arousal. All animals were allowed to recover and express sufficient opsin amounts for at least 3 weeks before testing.

### Optogenetic silencing

Before the experiment, mice were connected to the laser through a fiber optic cable (220 μm core diameter, 0.37 NA) and a fiber-optic rotary joint (Doric Lenses). To induce optical silencing of noradrenergic cells in the test group, yellow laser light at 589 nm from a DPSS laser (CNI Lasers) was used. Due to the sharp decline in activation of eNpHR 3.0 by red shifted light (Zhang et al., 2007), it is possible to use spectrally-close red laser light (658 nm) in the control group, instead of resorting to the use of non-functional optogenetic constructs. Such an EYFP-control would have required the use of an additional viral vector with different expression and membrane integration properties. The output power of the laser was adjusted to yield an illumination intensity of 10–15 mW/mm^2^ of light at the depth of the LC (Yizhar et al., 2011).

### Open field test

To test effects of LC suppression on locomotion, all animals were placed in an open field box (50 × 50 cm^2^, inner sector: 25 × 25 cm^2^) for 9 min. The animals were videotaped and the position of the animal was tracked with a custom program written in Matlab (TheMathworks). The open field test was performed three times during the experimental period. The first instance of the test took place 1 week before surgery. The second test was conducted 3 weeks after virus injection and in absence of light stimulation. These first two sessions served as habituation to the open field environment and to screen for a potential detrimental effect of surgery. The third test was conducted 2 days before the ASST. During this session, laser light-induced LC silencing was administered for 1 min each, after 2, 5, and 8 min post trial start, respectively (589 nm in all mice). During the third session behavior was analyzed separately for the time period of pre-silencing, during silencing and post-silencing, repeated three times each, to screen for potential effects of LC silencing on ongoing behavior or a rebound effects after LC silencing. The influence of optogenetic silencing on locomotion was measured by total distance and time spent inactive. As a measure of anxiety the time spent in the center section of the open field was detected.

### Attentional set-shifting task (ASST)

We used a modified version of the attentional set-shifting task (ASST; Bissonette et al., 2008) for reliable set shifting in mice. Since prior studies showed that single intra-dimensional discrimination was insufficient to form an attentional set in mice, we included additional presentations of the same relevant dimension (7 IDS stages) and an additional reversal stage after the compound discrimination (CDrev) to strengthen the formation of an attentional set. Furthermore, we only tested shifting from the dimension odor to digging material to guarantee a better comparability, because prior studies indicated a tendency for better performance when the shift was from the material to the odor (Kos et al., 2011). Using digging materials of different size and shape, but without a difference in material composition (chemically identical materials in both bowls), helped to avoid discrimination by odor and forces the animal to form an attentional set in the intended dimension.

**Figure 1:**
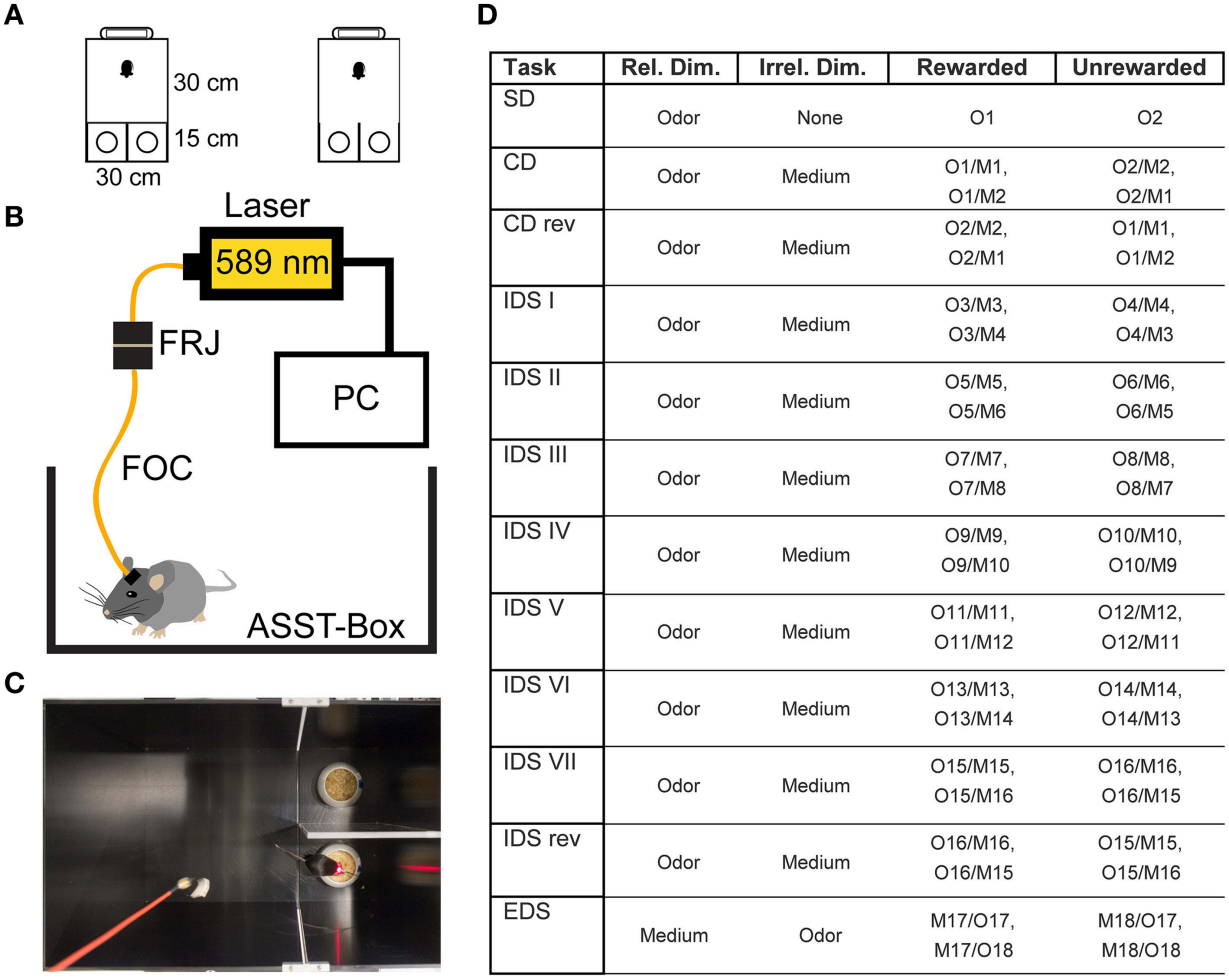
**Experimental Setup**. **(A)** Size and shape of the ASST box. The mouse was placed in the waiting compartment, the door was removed and the mouse allowed access to choice compartments. **(B)** Optical Setup. The mouse was connected to a fiber optic cable (FOC) that was connected to a fiber optic rotary joint (FRJ). Light was generated by a PC-controlled laser. **(C)** Example image of mouse in the box during control light illumination. **(D)** Table of odors and materials used for the different stages of the ASST: M1and2: wood pellets of different size, M3and4: aluminum foil pellets of different size, M5and6: cat litter of different size, M7and8: bark mulch of different size, M9and10: silica gel of different size, M11and12: plastic pellets of different size, M13and14: nuts and bolts, M15and16: metal shucks of different size, M17and18: gum Arabic of different size. Different olfactory stimuli were offered by various sweet dried herbs: O1, oregano; O2, parsley; O3, marjoram; O4, basil; O5, rosemary; O6, dill; O7, whitethorn; O8, stinging nettle; O9, lemon balm; O10, thyme; O11, ribgrass; O12, chamomile; O13, chives; O14, savory; O15, yarrow; O16, lime; O17, fennel; O18, mint.

The odors consisted of commercially available household spices mixed into the digging material (odors and materials used in each trial are tabulated in Figure 1D). Digging material always contained a small amount of reward powder to preclude an olfactory-guided search for the reward itself.

The box we used was similar to those used in previous studies (Colacicco et al., 2002). Briefly, the apparatus consists of three compartments. A waiting compartment (30 × 30 cm^2^) was separated by a Plexiglas door from two choice compartments (15 × 15 cm^2^, Figure 1A). Inside each choice compartment there was one digging bowl. One of the bowls was typically fitted with a reward (Kellog's Honey loop breakfast cereal). During the task, the mouse was allowed to transit from the waiting compartment to the choice compartments after the door was opened.

Before the main experiment, mice were habituated to the box over 3 days for two 10-min periods a day, spaced 3 h apart. During this time, the animals were free to explore and inspect the box and the bowls. During this habituation phase, both bowls contained a small piece of reward cereal. For habituation day 1, only the reward was placed in the bowls. For habituation day 2, the reward was placed on top of digging material. On habituation day 3 the reward was covered by digging material. As digging material during habituation home cage type litter was used. Under these conditions, mice learned quickly to dig for the reward. The session was terminated as soon as the mouse had found both food pieces or after 10 min elapsed, respectively.

During the ASST, only one of the bowls contained the reward. The two categories: digging material size/shape and odor were used to induce set formation. The ASST lasted 12 days. Each day, a different discrimination had to be performed (Figure 1D). The ASST started with a simple discrimination (SD), where only one dimension (the relevant dimension, two different odors) was presented in home cage type litter. On the next day compound discrimination (CD) followed, where the same relevant dimensions (odors) had to be discriminated, but two different digging materials were introduced as irrelevant dimension. The following day, compound discrimination reversal (CDrev) had to be performed. In this condition, reward contingencies are reversed, i.e., the previously unrewarded odor was now rewarded. Over the following 7 days of the task, intra-dimensional shifts (IDS I–VII) had to be performed. In these stages, different compound stimuli were presented, but the “relevant” dimension remained the same. In the course of these repetitions mice form an attentional set to attend to the relevant dimension (odor). After the last IDS, an intra-dimensional shift reversal (IDS VIIrev) was introduced, in which the reward contingencies of IDS VII were reversed, i.e., the rewarded and unrewarded odor are reversed. On the final day, mice had to perform the extra-dimensional shift (EDS), where new types of compound stimuli were presented, but the “relevant” dimension was changed (odor to digging material). Within the whole ASST, learning criterion was six consecutive hits. Not more than 31 trials per day were conducted, each of them lasting until the reward had been retrieved, the mouse started to dig in the wrong bowl or a maximum of 3 min had elapsed.

### Optogenetic silencing during the ASST

The optogenetic approach was based on a previous study (Carter et al., 2010). Briefly, to achieve optogenetic silencing of the LC, we connected the mice to a fiber optic cable (200 μm core diameter, 0.39 NA, Doric Lenses), attached to a fiber optic rotary joint (FRJ_1x1, Doric Lenses). The joint was connected to a 589 nm yellow laser (CNI) in the test group (*n* = 7) and a 658 nm laser in the control group (*n* = 7) (Figure 1B). Light application was controlled by a custom program written in LabView. Within each ASST trial, laser illumination was switched on at the start of the trial and switched off, when the trial ended or after a period of 1 min, whichever occurred first (Figure 1C). This limited illumination for 1 min was chosen over continuous light to avoid opsin desensitization. Twenty seconds after the start of illumination, the sliding door was opened and the mouse was allowed to enter the choice compartments.

### Histology

**Figure 2:**
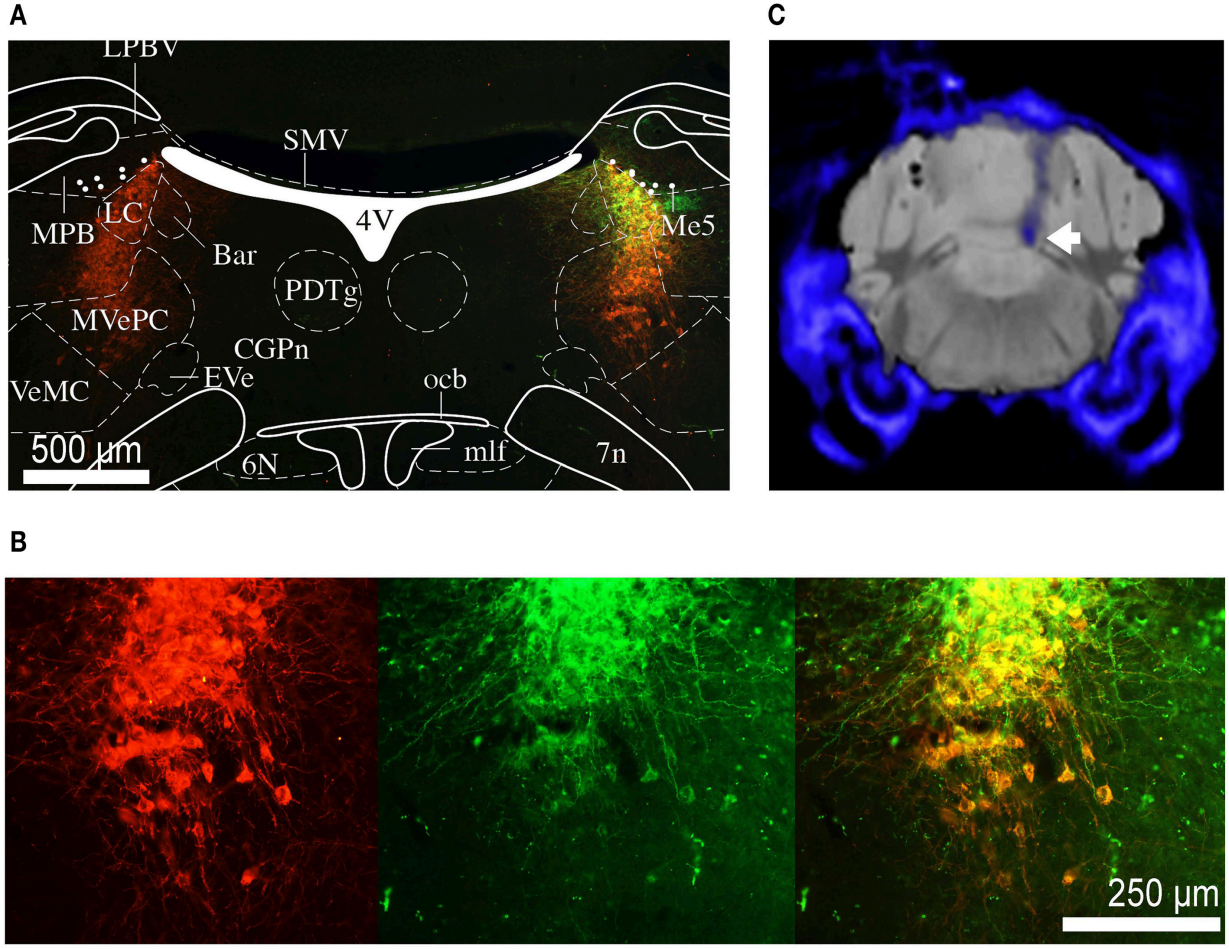
**Histology and micro CT**. **(A)** Section of the brain at the level of the LC. Red staining indicates TH positive neurons, green staining indicates eNpHR 3.0 opsin expression (through eYFP reporter). Depending on the level of opsin expression, the color if individual cells co-expressing TH and eNpHR 3.0 varies from orange to yellow. The overlaid atlas (Paxinos and Franklin, 2012) picture demonstrates that expression is restricted to the noradrenergic cells of LC. Labeled cell bodies outside the LC were not observed. **(B)** Magnified image of **(A)** showing co-labeling of TH (red) and eNpHR-eYFP (green). Scale bar: 500 μm. **(C)** Group average micro CT image of skull and fiber (blue) overlaid on standard mouse MRI dataset (gray). The tip of the fiber is placed directly above the LC (white arrow).

After completion of the experiments, the mice were perfused and brains removed for histology. The brains were sectioned into coronal slices (50 μm) and immunohistochemical analyses were performed. Noradrenergic cells were immune-stained with primary antibody against TH (polyclonal rabbit anti-TH ab112, Abcam). Anti-rabbit secondary antibody labeled with Alexa Fluor 546 was used. The expression of optogenetic construct and co-localization with TH-positive neurons was confirmed by fluorescence microscopy (Figures 2A,B). Due to the proximity of the LC and the ventricle and often poor tissue preservation, it was difficult to reconstruct the exact fiber position for all animals from histological sections. We have therefore resorted to micro-computed tomography (CT) scans, collected before perfusion, to confirm fiber position (Figure 2C).

## Results

### Open field

**Figure 3:**
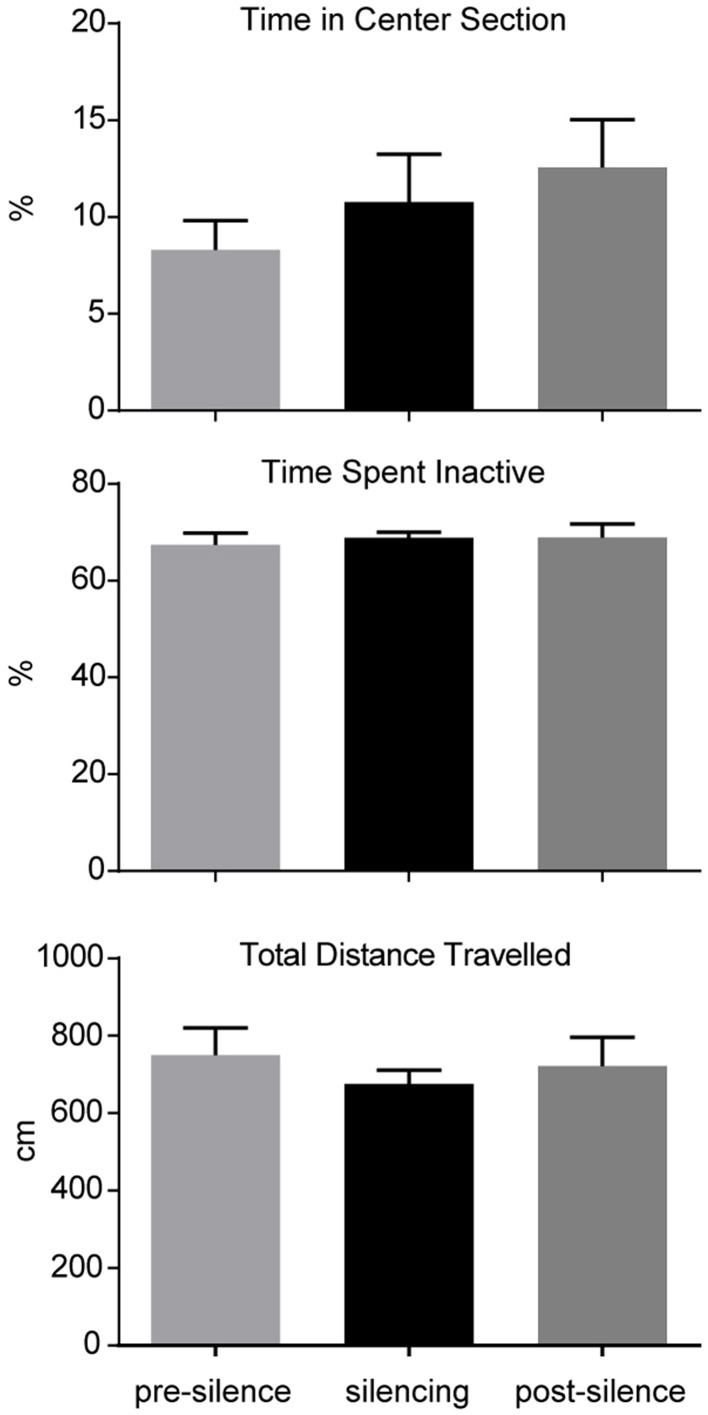
**Analysis of open field behavior during 1 min pre-silencing, silencing and post silencing for OF3**. The time spent in the center, time spent inactive and total distance traveled are not significantly affected by LC silencing. No significant difference exists between pre- and post-silencing periods that might potentially reflect a rebound of activity after silencing.

The influence of optogenetic silencing on locomotion was measured by total distance (pre-silence: 749.9 ± 70.8 cm; silencing: 675.2 ± 36.1 cm; post-silence: 722.4 ± 73.7 cm; *p* > 0.5, *F* = 0.36, One-way ANOVA) and time spent inactive (pre-silence: 67.4 ± 2.5%; silencing: 86.8 ± 1.2%; post-silence: 68.9 ± 2.8%; *p* > 0.5, *F* = 0.15, One-way ANOVA). As a measure of anxiety we calculated the time spent in the center section of the open field (pre-silence: 8.3 ± 1.5%; silencing: 10.8 ± 2.5%; post-silence: 12.6 ± 2.5%; *p* = 0.4, *F* = 0.95, One-way ANOVA, see Figure 3). These results show no significant locomotor or anxiety related effect of silencing. Furthermore, in the post-silencing period, no increased anxiety or locomotion over the pre-silenced period was evident.

### ASST

#### Impaired SD, CDrev and EDS by acute unilateral LC silencing

All mice readily performed the task despite being connected to the optical setup. Shortly after the sliding door was opened, mice entered the choice compartments and began exploring.

**Figure 4:**
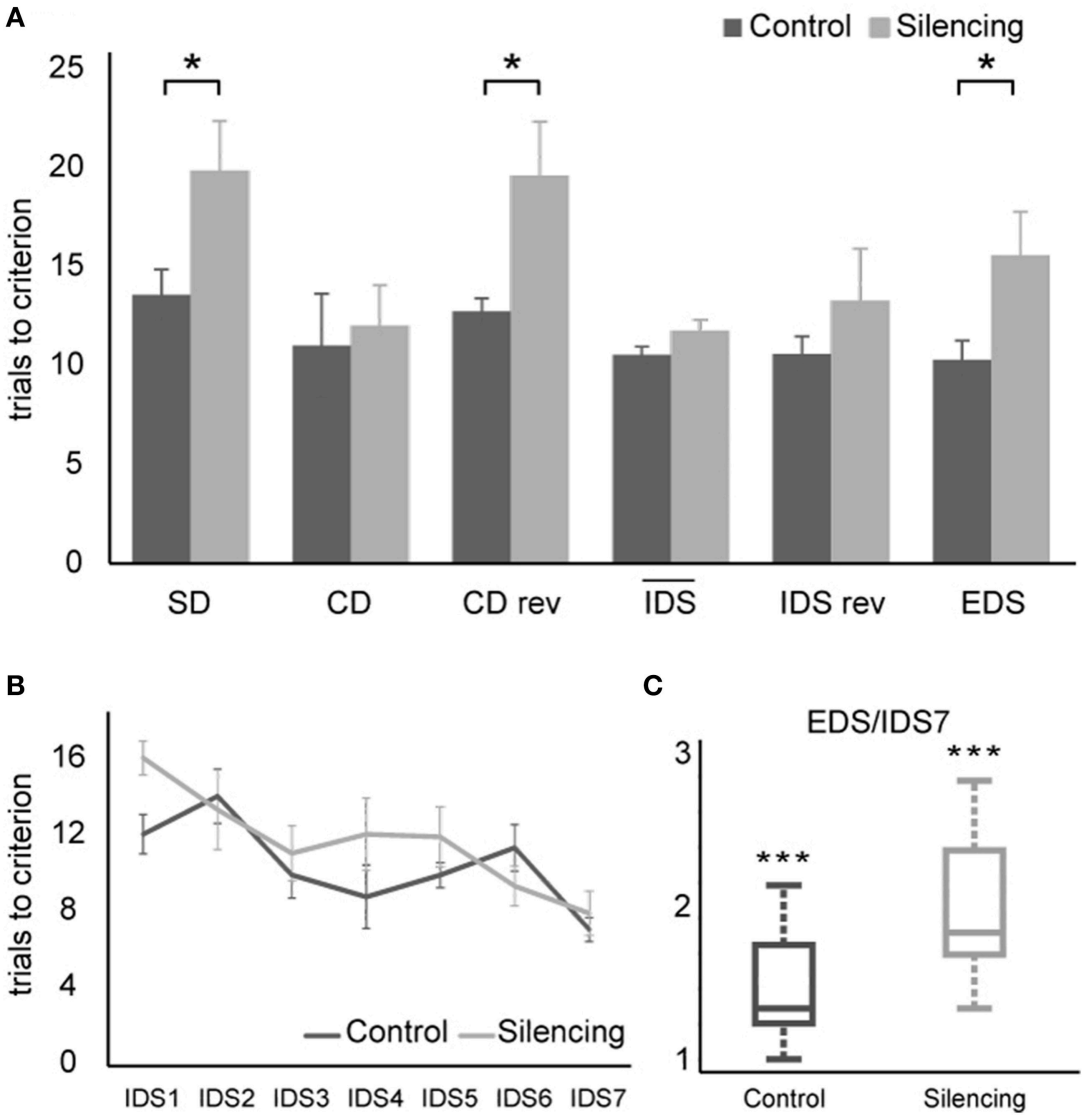
**Results of ASST**. **(A)** Average trials to criterion for simple discrimination (SD), compound discrimination (CD), compound discrimination reversal (CDrev), the mean across all seven intra-dimensional shifts (IDS), reversal of the last intra-dimensional shift (IDS VIIrev) and extra-dimensional shift (EDS). SD, CDrev and EDS were significantly impaired by optogenetic silencing. Due to the increased number of IDS stages in our paradigm, they were plotted separately in **(B)**. Average trials needed to criterion for all seven IDS conditions. Overall, the trials needed to reach criterion decreased over subsequent IDS conditions, indicating set formation. **(C)** Quotient between performance in the EDS and the IDS VII reflecting attentional “shift cost” after successful set formation. Values exceed unity significantly for both control group and silenced group, demonstrating that silencing did not interfere with set formation and that both groups successfully formed an attentional set.

A Two-way ANOVA with factors experimental stage and silencing revealed a significant effect of experimental stage and silencing [*F*_(11, 72)_ = 4.8, *p* << 0.01; *F*_(1, 72)_ = 18.7, *p* << 0.01, interaction *F*_(11, 72)_ = 1.8, *p* = 0.07]. For further analyses, we divided the task stages into two groups: one group most strongly relying on cognitive flexibility (SD, CDrev, EDS) and one containing the remaining stages that rely less on cognitive flexibility. In the group requiring cognitive flexibility, more trials to criterion were needed in the treatment group [18.3 ± 1.4 trials (mean ± S.E.M)] than in the control group (12.2 ± 1 trials). This effect was found to be highly significant (*t* = 3.6, df = 4, *p* = 0.01, one-sided unpaired *t*-test). In contrast, no significant effect of silencing was found in the group of experimental stages requiring less cognitive flexibility (silenced: 12 ± 0.8 trials; control: 10.6 ± 0.7, *t* = 1.33, df = 16, *p* > 0.1) (Figures 4A,B).

Successful set formation was validated by comparing the ratio of trials needed to reach criterion in the EDS as compared to the IDS VII stages. Both groups showed EDS/IDS VII ratios exceeding unity (control group: 1.44; treatment group: 1.94, *p* < 0.01 one-sided *t*-test; Figure 4C), indicating successful set formation.

During the extra-dimensional set shift (EDS), LC-silenced mice needed significantly more trials to criterion (15.6 ± 2.2, mean ± S.E.M.) than control mice (10.3 ± 1.0; *p* < 0.05 Welch-test). Comparable latencies to dig of LC-silenced and control mice during EDS performance (median in both groups: 32 s, mean ± S.E.M.: silenced 41.8 ± 8.9; control 35 ± 4.8, *p* = 0.5, Wilcoxon Rank-Sum Test) indicate that this difference was not caused by changes in running speed or response latency.

## Discussion

Several studies support the hypothesis, that the noradrenergic innervation of the PFC is critical for functions like attention and working memory (Devauges and Sara, 1990; Lapiz and Morilak, 2006; Chamberlain and Robbins, 2013). Furthermore, noradrenergic projections to the PFC might be critical for the ability to rapidly switch attention between stimuli and stimulus categories, leading to cognitive flexibility (Lapiz et al., 2007; Tait et al., 2007; McGaughy et al., 2008). Recently, optogenetic methods have started to allow a reversible manipulation of neuronal activity with high genetic and temporal precision. Optogenetic methods are well suited to selectively study the role of a small nucleus like the LC that can be targeted through a Th-Cre mouse line. The temporal fidelity of optogenetics allowed us to restrict silencing to a specific task phase, namely the acquisition phase, in order to investigate the effects of LC silencing in the context of a task that requires learning of new strategies and cognitive flexibility.

Due to the function of the LC for general arousal (Aston-Jones and Bloom, 1981; Lapiz and Morilak, 2006), we conducted initial open field experiments to assess potential side effects of the optogenetic silencing on locomotion. Changes in running speed or anxiety might have unspecific effects on ASST performance. Our results do not show a significant effect of unilateral silencing on locomotion. This supports previous observations, reporting that unilateral suppression of the LC has relatively few non-specific behavioral effects (Carter et al., 2010; Alsene and Bakshi, 2011). Importantly, after the cessation of silencing, no increase in locomotor activity over the pre-silenced period was evident. This finding suggests that if rebound firing in the LC occurs after light offset, it is likely weak and has little behavioral impact. This absence of generalized side effects makes optogenetic unilateral LC silencing especially suited for the study of cognitive functions in the context of a behavioral task. In contrast, stimulation has much more pronounced, general effects on locomotion that can overlap with task relevant functions or even lead to a complete behavioral arrest (Carter et al., 2010).

The used task, the ASST, is an animal analog of the ID/ED task and examines attentional set-shifting and reversal learning in mice (Owen et al., 1991; Garner et al., 2006; Bissonette et al., 2008; McGaughy et al., 2008). The ASST allows internal validation of attentional set formation by comparing the performance of animals in the last IDS vs. EDS stages. Poorer EDS vs. IDS performance would suggest that an attentional set to the initial relevant stimulus dimension was formed (Young et al., 2010). Attentional set formation is further indicated by improved performance from IDS I to IDS VII. Our results are in accordance to these requirements, hence it can be assumed that the mice did indeed form an attentional set in the silenced as well as control group. Successful set formation in the silenced group demonstrates that LC silencing does not impair general cognitive functions important for learning those parts of the task that are less dependent on cognitive flexibility. Set formation was even stronger in the silenced group as compared to controls, supporting our hypothesis that LC activity is mainly necessary during the acquisition of those parts of the task that require cognitive flexibility. An attentional set is a bias to attend to a particular stimulus dimension as a result of previous experience (Colacicco et al., 2002), and the cost of forming an attentional set is that it limits cognitive flexibility and therefore interferes with the ability to solve new inconsistent problems. Hence, during final EDS, when mice had to shift their attention to the prior irrelevant dimension that now predicts reward, they will take longer if the attentional set is more rigid or cognitive flexibility is reduced. Since, LC silencing during task performance did not generally impair learning but impaired EDS performance that requires cognitive flexibility, the “attentional shift cost” as ratio of EDS/last IDS trials is greater (Chase et al., 2012).

We found that silencing of the LC impaired performance particularly in those stages of the task that require cognitive flexibility, namely initial learning (SD), first reversal stage (CDrev) and extra-dimensional shift (EDS). During SD, the animal has to build a strategy to complete the task. Hence, a switch of attentional focus is required to establish a correct association between cue and reward. During CDrev, the learned strategy has to be abandoned and a new association has to be formed, and during EDS this association has to be performed even outside of the so far relevant stimulus dimension. This EDS stage, which required the highest cognitive flexibility, was also the most strongly affected stage in LC-silenced mice.

Different prefrontal subregions are required for these different cognitive functions. While reversal learning relies on an intact orbitofrontal cortex (OFC), the EDS requires mPFC- related functions. LC neurons are capable of modulating neuronal activity in each of these prefrontal subregions (Chandler and Waterhouse, 2012). Therefore, our present results are in accordance with lesion studies showing that selective noradrenergic deafferentiation of the mPFC impaired only EDS performance (McGaughy et al., 2008). Since LC-silencing modulates mPFC- as well as OFC-related functions, the found interference of silencing with reversal learning and set shifting is not unexpected. As demonstrated in the initial study from which we adapted the optogenetic method, LC silencing by eNpHR 3.0 results in a diminished norepinephrine release from LC terminals in the PFC (Carter et al., 2010). It is likely that this noradrenergic deficit is directly responsible for the observed effect on cognitive flexibility.

Interestingly, our results show an impact of LC silencing not only on EDS and CDrev performance, but also on SD performance. The found impairment in SD learning is in accordance with findings from mPFC-lesioned mice (Bissonette et al., 2008). This finding from Bissonette et al. was one further reason why we included SD into the cognitive flexibility group. Based on reported correlations between SD and EDS performance, a common mechanism might be responsible, for example the reduction of monoaminergic activity within mPFC, resulting in diminished cognitive flexibility (Colacicco et al., 2002). Data suggest that LC-silencing during task performance selectively interferes with acquisition, and inhibition of noradrenergic support in the absence of compensatory mechanism that are seen after lesions, induces deficits of initial learning and reversal learning in addition to impaired EDS. Therefore, our results strengthen the hypothesis that NE is recruited under conditions of unexpected uncertainty (Yu and Dayan, 2005; McGaughy et al., 2008). Due to the found effect on SD, EDS and reversal learning, it is likely that LC silencing is capable of modulating neuronal activity in different prefrontal subregions.

As mentioned above, prior studies based on lesions and pharmacological manipulations have already indicated that the noradrenergic LC is important for PFC-dependent functions. So far, irreversible lesion techniques or long lasting pharmacological manipulations were unable to differentiate between effects on acquisition phase or memory consolidation. Furthermore, after LC lesion with N-(2-chloroethyl)-N-ethyl-2-bromobenzylamine (DSP-4) functional recovery of LC noradrenergic neurons was reported by sprouting of the remaining noradrenergic axons that compensate the decreased noradrenaline in specific brain regions (Srinivasan and Schmidt, 2004). After DSP-4 lesion an increased concentration of NE was found in prefrontal cortex that indicates a gradual functional recovery (Srinivasan and Schmidt, 2004). A further post-lesion compensatory mechanism is that decreased NA levels in the PFC after LC lesion lead to changes in the adrenergic receptor profile and therefore influence spontaneous firing rate of mPFC pyramidal neurons (Wang et al., 2010).

The advantage of optogenetic silencing is that it is reversible and therefore no compensatory mechanisms are to be expected. Furthermore, it can be restricted to a particular phase alone and does not interfere with the functions of the LC during other times.

The shown interference of LC silencing with the acquisition phase of the task is in accordance with prior studies investigating reversible bilateral functional inactivation of LC by means of stereotaxic local microinjection of lidocaine. Khakpour-Taleghani and colleagues also show significantly impaired acquisition of spatial memory, but no effect on consolidation and/or retention in the Morris water maze task (Khakpour-Taleghani et al., 2009). Therefore, our results support the hypothesis that the noradrenergic system of the LC may play a more important role in acquisition than in consolidation and retrieval of memory.

Making use of the ASST, our study helps to more precisely define the specific role of the LC in frontal cortex dependent functions. From the results of this study, it is not possible to conclusively attribute the found effects to a particular frontal region. Based on the variety of projections it seems likely that the LC modulates larger parts of the frontal network. A more detailed study testing local silencing of LC terminals in various frontal regions could therefore provide more detailed information about the regions that are affected and under which circumstances they are recruited. In summary, our study demonstrates a specific influence of LC function on the acquisition phase of ASST stages that strongly rely on cognitive flexibility. Based on the anatomical projection pattern of the LC, this influence likely originates in the diverse network of frontal cortex and its noradrenergic LC-projections.

## Conflict of interest statement

The authors declare that the research was conducted in the absence of any commercial or financial relationships that could be construed as a potential conflict of interest.
